# Duration of symptoms in the quantification of upper limb disability and impairment for individuals with mild degenerative cervical myelopathy (DCM)

**DOI:** 10.1371/journal.pone.0222134

**Published:** 2019-09-09

**Authors:** Sukhvinder Kalsi-Ryan, Jerri Clout, Pouya Rostami, Eric M. Massicotte, Michael G. Fehlings

**Affiliations:** 1 Toronto Rehabilitation Institute, University Health Network, Toronto, Ontario, Canada; 2 Department of Physical Therapy, University of Toronto, Toronto, Ontario, Canada; 3 Spine Program, Toronto Western Hospital, University Health Network, Toronto, Ontario, Canada; 4 Department of Surgery, University of Toronto, Toronto, Ontario, Canada; University of Utah Hospital, UNITED STATES

## Abstract

**Objectives:**

Degenerative cervical myelopathy (DCM) involves spinal cord compression, which causes neurological decline. Neurological impairment in DCM is variable and can involve complex upper limb dysfunction including loss of manual dexterity, hyper-reflexia, focal weakness, and sensory impairment. DCM can cause progressive loss of manual dexterity, reduced upper limb (UL) function and disability. The purpose of this study was to define relationships between impairment and disability of the UL and determine the impact of duration of symptoms on disease severity.

**Design:**

An observational cross-sectional study quantifying disease severity, UL impairment and disability at time of diagnosis was conducted. A second observational longitudinal cohort was studied at the time of diagnosis and 1 year later.

**Setting:**

Toronto Western Hospital, Spine Program.

**Subjects:**

The cross sectional study included 140 study subjects diagnosed with mild, moderate or severe DCM. For the longitudinal study, 61 study subjects with mild DCM were enrolled and split into two groups, one group with less than 12 months of symptom duration and more than 12 months.

**Main measures:**

Modified Japanese Orthopaedic Assessment (mJOA); Graded Redefined Assessment of Sensation, Strength and Prehension (GRASSP); *Quick* Disability of the Arm, Shoulder and Hand (*Quick*DASH).

**Results:**

Pearson correlation coefficients between GRASSP and *Quick*DASH revealed significant relationships between strength, sensation and dexterity for all patients to varying degrees. The covariate (mJOA) was significantly related to *Quick*DASH, indicating duration of symptoms has an important effect on UL disability in the mild severity group.

**Conclusions:**

Strength, sensation and dexterity play a defining role in disability of the UL across all severities of DCM and are discriminant measures. Duration of symptoms has a significant impact on self-perceived disability, where a longer duration in mild patients results in diminished disability, suggesting adaptation. Duration of symptoms is an important factor to consider in the treatment plan for patients with mild disease.

## Introduction

Degenerative Cervical Myelopathy (DCM) encompasses the chronic progressive compression of the cervical spinal cord due to degenerative disc disease, spondylosis, or other degenerative spinal pathologies. It is the most common form of spinal cord impairment and can cause functional decline leading to reduced independence and quality of life [1–4]; with a main consequence being loss of manual dexterity related to reduced upper limb (UL) function and disability. To date, the modified Japanese Orthopaedic Assessment (mJOA) has been used to quantify the severity of disease into mild, moderate or severe symptoms. Typically, patients with moderate or severe presentation of DCM are offered surgical decompression, which often results in improved functional status [5,6]. However, for patients presenting with mild DCM, surgical intervention is not recommended [7]. Instead, a period of watchful waiting and non-operative management is the current standard of care [8].

Mild DCM describes a neurological deficit related to minimal spinal cord compression and reduced blood flow to the cord [9]. The symptoms are often subtle, such as: some numbness or tingling of the hands and/or arms, neuropathic symptoms of pain in the arms, mild instability of gait, reported minimal difficulty of fine motor tasks and rare bladder symptoms. Imaging reveals varying degrees of compression, which can look mild or significant, however, if the clinical presentation is not consistent with the imaging, surgical intervention is not a clear cut option [5, 8]. Typically the neurological signs are also mild where strength and sensation are assessed as intact with possible hyper-reflexia of the upper limbs and/or a positive Hoffman’s sign. Neurological symptoms and signs, along with imaging, define a picture of DCM, however, this information is not always useful when making decisions regarding disease management. For this reason, the management of mild DCM is controversial and balancing the risks/benefits of surgery or monitoring disease progression becomes the dilemma.

Improved ability to quantify impairment and disability may assist in understanding the disease and its sequelae; which could ultimately lead to a more informed management of mild DCM. In this study, we evaluated both impairment and disability to understand the relationships between these two domains and the role neurological impairment plays in disability. Current methods of assessment in DCM have not addressed impairment or disability adequately [6], or defined the impact of the duration of symptoms on UL disability. It is known that duration of symptoms prior to surgery can predict post-operative outcome for patients with moderate to severe DCM [10]. However, the mJOA is not sensitive enough to detect subtle changes in the mild sub-group and a concrete understanding of this phenomenon, using validated impairment and self-reported disability measures, has not been documented. This study takes the novel approach of using the *Quick* Disabilities of the Arm Shoulder and Hand (QuickDASH) [11, 12], a measure of self-perceived upper limb disability, and relates it to deficits in sensation, strength and dexterity. The objectives of this work were to 1) define the relationships between impairment and disability of the upper limb for patients with DCM; and 2) explore the relationship between duration of symptoms and disability of the UL across severity sub-groups.

The authors hypothesized that there would be relationships between impairment (domains) and disability, however, the relationships would vary across severities. With respect to self-perceived disability, the authors hypothesized that disability would only be impacted by degree of impairment and no other factor.

## Materials and methods

### Participants

The University Health Network Research Ethics Board provided ethics approval for the study. Patients presenting with DCM at the Toronto Western Hospital were prospectively screened, given study details, and potential study participants then provided written informed consent for inclusion in this study. Patients were included if: they were able to provide informed consent, were between the ages of 18 and 85, had a diagnosis of DCM (the presence of at least one clinical symptom and one neurological sign and a positive MRI) [4, 7]. Patients were excluded if: they were unable to communicate in English, were over 85 years old, had a duration of symptoms greater than ten years, or were unable to provide informed consent.

### Study design

A cross-sectional, observational study was conducted from 2008–2014 on a prospectively accrued sample of 140 individuals with DCM. All patients were assessed following their initial neurosurgical consultation but prior to therapeutic intervention (operative or non-operative management). Demographical information, including age, gender and duration of symptoms, a detailed neurological exam, modified Japanese Orthopaedic Assessment Scale (mJOA) [6, 13], the *Quick*DASH [11, 12], and a modified version of the Graded Redefined Assessment of Sensation, Strength and Prehension (GRASSP) [12, 14] were collected for each individual.

A second prospective, longitudinal (2014–2016) cohort study of 75 patients was conducted to further assess the affect of duration of symptoms on perceived disability and impairment. Fourteen patients were lost to follow up, and of the remaining 61 patients in this cohort, 25 presented with mild DCM for less than one year at baseline while 36 patients presented with mild DCM for more than one year at baseline. All patients were assessed at baseline and one year after enrollment in this longitudinal cohort.

### Outcome measures

The WHO International Classification of Functioning (ICF) domains were used to select outcomes implemented in this study to assess patient impairment and disability [15].

#### Modified Japanese Orthopaedic Assessment Scale (mJOA)

The mJOA is a frequently used outcome measure to assess DCM patients [12], and was used to stratify the study population according to disease severity [4, 10]. Validity of the mJOA has been established, internal consistency is defined by a Cronbach’s alpha of 0.630 and external consistency with the Nurick is r = 0.625. Responsiveness was also defined and shows a Cohen’s effect size of 1 over a 12 time course [6]. Despite the popularity of the mJOA, it is a whole body measure and is not sufficiently sensitive to understand UL impairment alone. Furthermore, it has been noted that the mJOA is not sensitive to subtle changes in mild DCM patients before and after surgical decompression [10].

#### Graded Redefined Assessment of Strength, Sensation and Prehension (GRASSP)

The GRASSP has proven to be sensitive to UL function and useful in quantifying neurological impairment in traumatic spinal cord injury [15]. In addition to defining neurological function, the GRASSP also characterizes the compensatory behaviours resulting from gradual neurological decline. Here, the sensory, strength and dexterity elements from the GRASSP were used to quantify UL impairment in DCM [14], as the prehension elements are not sensitive to mild DCM impairment.

#### Quick Disability of the Arm, Shoulder and Hand (QuickDASH)

The *Quick*DASH was designed to measure physical disability of any region of the UL originally for work related repetitive strain injury. It has been proven to be a valid, reliable measure with good sensitivity to changes in condition [11]. This study is the first to characterize the use of the *QuickDASH* as a measure of UL disability in the DCM population.

### Analysis

Patients were stratified according to the severity of disease using the mJOA. Disease severity was categorized as mild (mJOA = 17 to 15), moderate (mJOA = 12 to 14) or severe (mJOA < 12). The mJOA stratification followed previously defined cut points of the DCM population (5). A second stratification was performed to be more specific in relation to the UL (mJOA UL), as previously characterized [16]. The UL sensation and UL motor subscores of the mJOA were summed and used to stratify the sample for the analysis of duration of symptoms to ensure the deficit was specific to the upper limb. The maximum summed mJOA UL score is 8 (normal UL function), and the stratification used was as follows: mild (mJOA UL = 6 and 7), moderate (mJOA UL = 4 and 5) and severe (mJOA UL = 1–3).

To examine the relationship between impairment (GRASSP subtest scores of strength, sensation and prehension/dexterity) and disability (*Quick*DASH scores), Pearson correlation coefficients within each severity group were defined. All tests of significance were two tailed and significance level was set as p ≤ 0.05.

The effect of duration of symptoms on disability was studied in 87 patients (complete data was unavailable for the remaining 53 cases). An ANCOVA was conducted controlling for mJOA UL to determine the effect of duration of symptoms on disability. The *Quick*DASH, used as a measure of disability, was plotted against duration of symptoms and the mJOA UL score was used to stratify according to severity of disease. T-tests were used to compare patients with a duration of symptoms of less than a year to patients with a duration of more than a year across all severity subgroups.

## Results

### Sample

A sample of 140 individuals (81 males and 59 females) were enrolled in the cross sectional study. The mean age of the population was 57.68 +/- 12.63 years and the mean duration of symptoms was 3.6 +/- 4.2 years. Thirty one (17% (X-15.5, SD-0.7 [Range: 15–17]) subjects presented with a mild severity according to the mJOA, 57 presented with moderate (41% (X-13.1, SD-0.8 [Range: 12–14]) and 59 presented with severe disease (42% (X-9.4, SD-1.4 [Range: 5–11]). Tables 1 and 2 define the sample by total mJOA and mJOA-UL severity stratification with group means and standard deviation for each measure administered, respectively. It should be noted that all patients enrolled presented with at least mild DCM, therefore, the highest mJOA score obtainable was 17 meaning that the mJOA did not capture UL deficit, or in some cases there is a possibility that only lower limb deficits were present.

**Table 1:** Means and standard deviations for each severity group as defined by the mJOA.

Severity	n	GR-sens (0–24)X (SD)	GR-str (0–100) X (SD)	GR-pp (sec) X (SD)	*Quick*DASH(0–100) X (SD)
Mild	59	23.16 (1.97)	98.13 (2.35)	93.96 (43.57)	18.51 (17.79)
Moderate	57	20.78 (3.62)	94.83 (5.17)	116.00 (36.06)	38.81 (22.03)
Severe	31	17.62 (5.87)	88.32 (13.12)	133.05 (57.49)	57.12 (19.93)

mJOA score of 17–15 = mild, 14–12 = moderate, less than 12 = severe [5]; GR-sens = GRASSP Sensation, GR-str = GRASSP Strength, GR-pp = GRASSP Prehension Performance.

**Table 2:** Known groups validity—*Quick*DASH features stratified by mJOA-UL scores.

mJOA UL Severity	*Quick*DASH mean	*Quick*DASH SD	*Quick*DASH Range
Normal	11.65	19.87360058	0–50
Mild	30.76865672	21.62407615	0–50
Moderate	54.66521739	18.91398327	11.4–86.4
Severe	62.4	22.15779772	18.2–100

mJOA-UL score of: 8 = normal, 6 or 7 = mild, 5 or 4 = moderate, 3–1 = severe; SD = Standard Deviation

### Validity of QuickDASH and Relationships between Impairment and Disability

Tables 3 and 4 present the statistical relationships between the sensation, strength and prehension/dexterity portions of the GRASSP with scores on the total mJOA and mJOA UL, respectively, for all severity groups. As UL impairment (GRASSP) increases, disability (*Quick*DASH*)* also increases. The impairment profile of the disease changes with increasing disease severity (Table 2). Thus, the degree to which sensation, strength and dexterity play a role in UL disability varies according to the severity of the disease.

**Table 3:** Relationship between QuickDASH and clinical measures of upper limb impairment after stratification of the sample using total mJOA scores.

	Mild N = 59		Moderate N = 57		Severe N = 31	
	*r*	*p*	*r*	*p*	*r*	*p*
Quick DASH/GR-sens	-0.347	0.01	-0.102		-0.058	
Quick DASH/GR-str	-0.574	<0.001	-0.361	0.005	-0.481	0.006
Quick DASH/GR-pp	0.285	0.038	0.043	0.029	0.072	

mJOA score of: 17–15 = mild, 14–12 = moderate, less than 12 = severe [5]; GR-sens = GRASSP Sensation, GR-str = GRASSP Strength, GR-pp = GRASSP Prehension Performance; r = Pearson Correlation Coefficient; p = p-value. A significance level of p<0.01 was used.

**Table 4:** Relationship between *Quick*DASH and clinical measures of upper limb impairment after stratification of sample using upper limb mJOA-UL scores.

mJOA-UL	Normal (n = 8)		Mild (n = 71)		Moderate (n = 49)		Severe (n = 12)	
	r	p	r	p	r	p	r	p
Quick DASH/GR-sens	-0.037		-0.226	<0.001	-0.367	0.009	-0.276	
Quick DASH/GR-str	-0.798	0.01	-0.355	0.002	-0.416	0.002	-0.741	0.005
Quick DASH/GR-pp	-0.421		0.089		0.316	0.02	0.433	AS

mJOA-UL score of: 8 = normal, 6 or 7 = mild, 5 or 4 = moderate, 3–1 = severe; GR-sens = GRASSP Sensation, GR-str = GRASSP Strength, GR-pp = GRASSP Prehension Performance; r = Pearson Correlation Coefficient; p = p-value. A significance level of p<0.01 was used.

### Relationship between duration of symptoms and disability

The covariate (mJOA UL) was significantly related to the *Quick*DASH, F (1, 58) = 6.939, p = 0.011. Final results indicated duration of symptoms had a significant effect on *Quick*DASH scores, F (27, 58) = 1.831, p = 0.027

To determine the effect of duration of symptoms on patient disability, an ANCOVA was conducted on the data pertaining to the patients with mild (6–7), moderate (4–5), or severe (0–3) mJOA UL scores. While significant relationships were shown for the mild group (F (16, 27) = 4.934, p < 0.0001), no relationship was seen for moderate, (F (21, 9) = 1.704, p = 0.207) or severe groups (F (3, 1) = 0.805, p = 0.654).

Using an independent-sample t-test, the mJOA UL scores of those with a symptom duration of less than a year (Group A) were compared to scores of those with a symptom duration of more than a year (Group B). A significant difference was seen between the scores of Group A (M = 5.7436, SD = 1.18584) and Group B (M = 26.6625, SD = 28.41757) (t (47.201) = 5.095, p < 0.0001).

The same analysis was then conducted on the mild, moderate, and severe mJOA UL severity groups separately. In the mild sample, scores differed significantly between Group A (M = 6.500, SD = 0.51177) and Group B (M = 13.5261, SD = 15.11394) (t (22.053) = 2.228, p = 0.036). A similar variance was seen in the moderate sample between Group A (M = 4.5714, SD = 0.51355) and Group B (M = 38.1222, SD = 30.35168) (t (17.013) = 4.689, p < 0.0001). However, the severe sample did not show a significant difference between Group A (M = 5.5000, SD = 3.53553) and Group B (M = 60.4000, SD = 41.17572) (t (4) = 1.776, p = 0.150).

### Longitudinal evaluation of duration of symptoms, a follow up cohort

**Fig 1:**
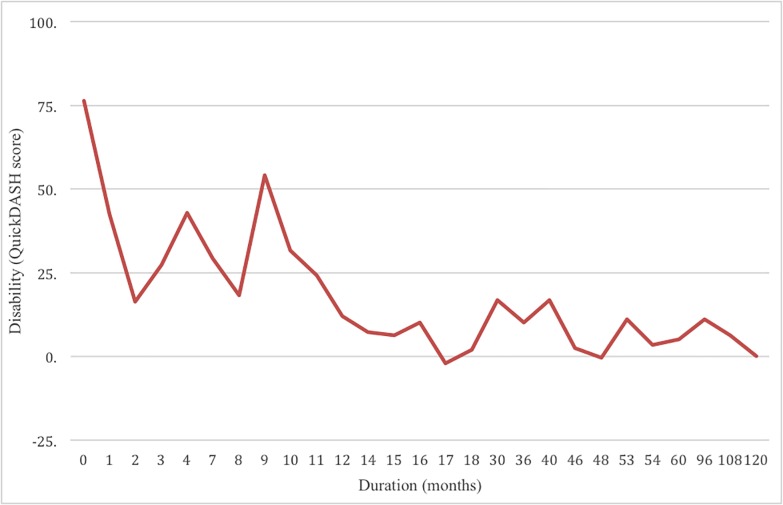
The relationship between self-reported disability and the duration of symptoms. QuickDASH scores for a cross section of patients against duration of symptoms in months. There is a notable decline in perceived disability in the two groups (Group 1 less than 1 year of duration versus Group 2 more than 1 year duration). This unexpected finding led the investigative group to look closer at other variables that might behave in this manner in a prospective study. Note that the duration axis is not uniform.

Subtle changes in manual dexterity were examined in patients with a duration of symptoms either less than or greater than one year. It was hypothesized that during the early stages of DCM (<12 months symptoms), patients with mild DCM would present with more pronounced self-perceived disability and greater changes in hand function. This hypothesis was supported by the analysis of the first study cohort, where individuals with DCM for less than a year reported greater disability as compared to those who had DCM symptoms for more than a year (Fig 1). The results and differences between both groups are defined in Tables 5 and 6.

**Table 5:** Sample characteristics (n = 61) of follow up study group.

Group	n	Female:Male	Age X (SD)	# Neurological Symptoms X (Range)	# Neurological Signs X (Range)
Group 1	25	8:17	56 (10)	2.8 (0–6)	1.56 (0–4)
Group 2	36	16:20	55 (12)	2.9 (0–6)	1.51 (0–6)

Group 1 –Duration of symptoms less than 12 months; Group 2 –Duration of symptoms greater than 12 months

**Table 6:** GRASSP Assessment results for Group 1 (duration of symptoms <12 months) and Group 2 (duration of symptoms >12 months).

	BL mJOA	FU mJOA	BL QD	FU QD	BL Str	FU Str	BL Sens	FU Sens	BL Dex	FU Dex
**Group 1R X (SD)**	15.9 (0.8)	15.5 (1.1)	30 (13)	18 (12)	48(3)	49(1.5)	10(2)	11(1)	**6.6/56**	7.3/50
**Group 1L X (SD)**					47 (3)	47(3)	11(1.5)	11(1)		
**Group 2R X (SD)**	15.9 (0.8)	16.0 (1.6)	20 (17)	27 (20)	48(2)	48(2)	11(1)	11(1)	**7.4/50**	6.6/53
**Group 2L X (SD)**					48 (2)	46(5)	11(1)	11(1)		

Group A—mild DCM for less than a year; Group B—mild DCM for greater than a year

BL—baseline; FU—follow-up; Str–GRASSP strength (0–50); Sens–GRASSP sensation (0–12); Dex–GRASSP Dexterity (0–9); R–Right; L–Left.

It is noted that manual dexterity and upper limb disability is more pronounced in Group 1 (DCM for less than a year) at baseline. Dexterity is more consistent with presentation that reflects moderate severity. However, dexterity improves one year later to be consistent with expected mild DCM presentation. It is also noted that Group 2 (DCM for more than a year) presents with dexterity and disability deficits which are more consistent with expected values of mild DCM, however, over one year these patients show subtle deterioration.

## Discussion

This work aimed to explore and better understand the presentation of DCM with outcomes that focus on disability and specific impairments of the hand. Here, we demonstrate subtle elements of disease presentation and provide validation of tools that can be used to characterize DCM and aid in clinical decision-making. These measures show good validity in discriminating DCM and are sensitive to changes that are not typically captured during a conventional neurological exam.

### Relationship between impairment and disability

The degree of UL impairment was positively correlated to the level of disability in patients with DCM. Disability is specifically caused by impairments in sensation, strength, and/or prehension. The contribution of each impairment domain to disability is dynamic as the severity of the disease progresses. Patients with mild DCM have disability caused by impaired sensation, strength and dexterity, whereas patients with severe DCM tend to have disability largely attributable to impaired strength and dexterity.

### Validity of QuickDASH as a measure of Disability in the DCM population

Based on the relationship between impairment and disability, the *Quick*DASH is a valid tool to define UL disability in the DCM population. The impairment and disability measures used in this study are also capable of discriminating severity within the DCM sample and can facilitate understanding of the underlying impairments and causes of disability. Thus, it can be stated that elements of the GRASSP and the *Quick*DASH hold concurrent validity with the mJOA and can be used as ancillary outcome tools within the DCM population. Use of more than one outcome measure is important because the variability of the disease is so great that one measure alone cannot characterize the multiple factors that cause disability [12, 17, 18].

UL disability for patients with mild DCM was found to be influenced by all three domains of impairment: sensation, strength, and dexterity. However, the analysis defined disability associated with moderate and severe DCM to only be influenced by strength and dexterity. This is likely because individuals with moderate or severe DCM present with more profound motor and dexterity deficits; however, one underlying cause of dexterity impairment is sensory impairment [19]. Our results indicate that it is critical to consider quantitative sensory impairment in addition to measuring strength and dexterity in the neurological assessments of mild DCM patients, as loss of sensation is the primary complaint among these patients [20] and early in the disease progression reduced sensation is the main factor contributing to UL disability. A similar set of relationships is seen when the group is stratified by UL mJOA, however, sensation plays a significant role in both mild and moderate severity with this classification of the mJOA [16]. All three components of impairment remained significant through the mild and moderate ranges of severity, again leaving strength as the single significant factor at the severe stage. The shift in significance of impairment is likely due to the addition of other factors of DCM that contribute to severity, such as pain, bladder dysfunction, and lower extremity involvement.

### Relationship between duration of symptoms and disability

Duration of symptoms plays an important role in defining disease presentation and severity. A longer duration of symptoms predicts a smaller self-perceived disability when controlling for impairment in mild DCM patients (Fig 1). This is likely due to mental and physiological adaptations that occur as one learns how to accommodate the physical changes caused by cord compression [8, 9]. In this study, disability began to diminish at 9 months, and by 12 months disability was greatly reduced. As a result of these findings, the duration of symptoms associated with a mild presentation of DCM is a critical factor. A duration of 12 months or more indicates that physiological and/or psychological adaptation has likely occurred. The mJOA may not be sensitive to changes in the mild DCM patient because it does not capture the subtle sensory, motor and dexterity changes that are fluctuating while the patient is adapting neurologically. Thus in practical terms, patients presenting with less than 12 months of mild DCM can present with greater deficit in dexterity and self reported UL disability than patients presenting with mild DCM for longer than one year. During the first year the patient adapts to the cord compression and is able to have a fluctuating presentation of disease, but remains in the mild range. We can confirm that this phenomenon is not only psychological, but rather a combination of psychological and physiological change that occurs as a result of neuroplasticity.

These findings provide some insight into how the disease presents early on and how the patient adapts to the neurophysiological phenomenon of cord compression. There are subtle improvements in hand dexterity and significant improvement in disability throughout the first year. After 12 months, the mild symptoms persist and likely stabilize. We suspect that patients have a period of mild presentation that fluctuates for an unknown period of time, and subtle changes in function can be noted with more sensitive tools to show that the disease is progressive in very small increments, causing greater deficit each year. The duration of symptoms for patients with mild DCM is a critical factor in determining management. The first year of presentation should be a period of close observation for mild DCM patients. If presentation and symptoms persist beyond one year, the severity and intensity should be assessed to determine if there is any progression of disease. If there is no obvious progression it is important to note that there is cord compression and moving forward the patient will deteriorate on a yearly basis. In this situation, surgical intervention should be offered when the patient begins to transition into the moderate severity [21]. The presentation of a mild patient is not static and management of this subgroup remains controversial. Patients go through a period of suspected neuro-adaptation as they learn to live with symptoms in the first 12 months of DCM. Following the first year of symptoms, patients stabilize and the eventual progression is not well understood. However, there is a concern as to when and how much mild DCM patients will decline.

The *Quick*DASH is useful when administered in conjunction with an impairment measure as a screening tool in order to define UL disability in DCM. This tool defines for the clinician the patient’s perspective of how disabling the condition is, which can have an impact on the clinician’s decision. When using *Quick*DASH, one should always consider the effect that the duration of symptoms has on the results of self-reported disability, especially among those with mild symptoms. Consequently, these findings influence the clinician to consider a timely intervention, which may be very important for the successful management of this disease [8].

Limitations of this work are related to the results and conclusions being based on a cross section of data and small longitudinal sample. Furthermore, this work does assist in placing the understanding and management of DCM among more common diseases such as lower back pain and lateral epicondylitis through characterizing this sample with both novel and sensitive impairment and disability measures.

## Conclusions

This work confirms a number of concepts for clinical application. First, there is a greater understanding of the presentation of UL disability when the underlying impairment is defined. Both the *Quick*DASH and elements of the GRASSP are valid and useful ancillary measures to define DCM and the severity of disease to ultimately inform clinical decisions regarding management. Secondly, the duration of symptoms is a significant clinical indicator that should be accurately defined and considered in clinical decision making. Mild patients can present as improved or stable after one year from first symptoms, which makes the management controversial. Duration of symptoms is an important factor to consider when planning treatment for mild patients, particularly when considering the timing of surgical intervention. While surgical intervention is not a common management approach for mild patients, baseline and observational assessments will be determinants of the approach. For mild DCM individuals that are deemed non-surgical candidates, education regarding DCM, symptoms and progression can be useful in addition to close observation from a clinician. Education and reassurance regarding symptoms elevates awareness for patients and empowers them to recognize red flags if they arise.

Considering the controversial nature of how to manage mild DCM, this work elucidates some of the adaptations patients encounter in the first year. Importantly, mild DCM patients should not only receive close observation, but rather, a decision to manage this population with observation or surgery can depend on the patient’s self-perception of deficits. While it is relatively atypical to encounter a mild DCM patient with less than 12 months of symptomology, this work provides insight on the behaviour of the disease and expands on knowledge needed to make a treatment decision. Thus, the duration of symptoms and clinical presentation need to be carefully considered when planning management for the mild DCM patient.

## Supporting information

S1 Dataset Raw data. (XLSX)
